# Shifting growth–climate limitations in Canadian forests under recent climate change

**DOI:** 10.1038/s43247-026-03713-7

**Published:** 2026-06-26

**Authors:** Zihaohan Sang, Andreas Hamann, Martin P. Girardin, Vinicius Manvailer

**Affiliations:** 1 Department of Computer Science, University of Toronto, Toronto, ON Canada; 2 Department of Renewable Resources, University of Alberta, Edmonton, AB Canada; 3 Laurentian Forestry Center, Canadian Forest Service, Natural Resources Canada, Quebec City, QC Canada; 4 Pacific Forestry Center, Canadian Forest Service, Natural Resources Canada, Victoria, BC Canada

**Keywords:** Climate-change ecology, Evolution, Forest ecology, Boreal ecology

## Abstract

Productivity of northern forests is often expected to increase with warming, but rising atmospheric demand for water may offset these gains. Here we analyze 38,395 tree-ring chronologies from 4179 Canadian sites over 120 years to quantify how growth limitations respond to climate change. In the mid- to late 20th century, drought limitations already dominate nearly half of Canada’s forested area, while cold limitations are largely restricted to high-latitude and montane regions. Examining changes in growth limitation through time, we identify four response modes across regions: persistence, shifts from cold to drought limitation, reversals toward cold limitation after mid-century drought sensitivity, and stable cold limitation in wet hydroclimates. These patterns show that forest growth responses are not gradual, but can cross thresholds or reverse under continued warming. The results suggest that responses to warming may not translate into sustained gains across large areas of northern forests, including many commercially important regions.

**Figure:**
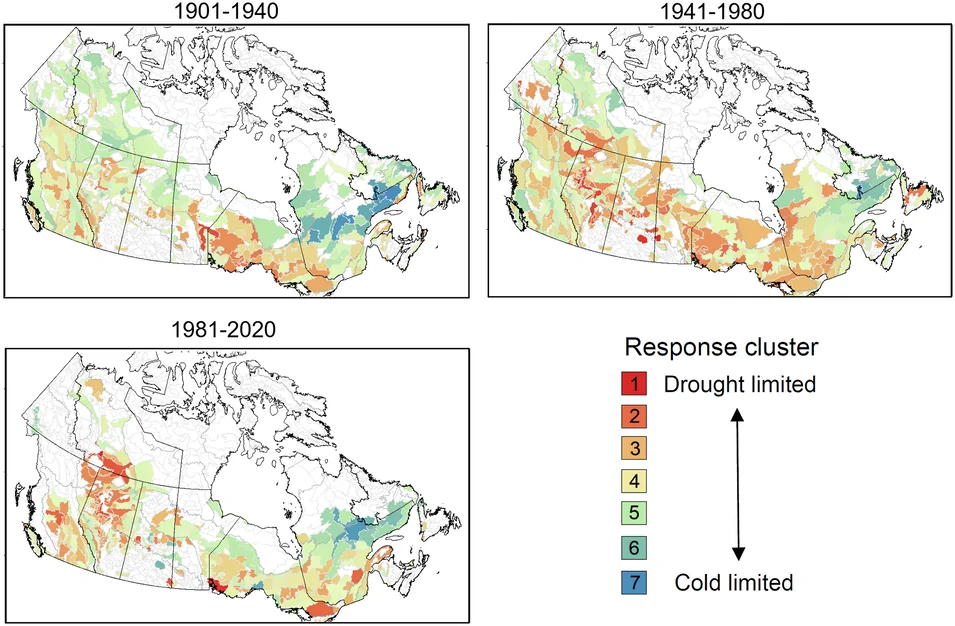

## Abstract (web summary)

Drought stress already constrains much of Canada’s forests, with warming pushing growth across moisture thresholds where limitations can persist, shift from cold to drought, reverse, or remain buffered by regional hydroclimate, based on 38,395 tree-ring chronologies from 4,179 sites.

## Introduction

High-latitude ecosystems, including Canada’s extensive boreal forests, have long been expected to benefit from climate warming because reduced cold stress and longer growing seasons should enhance productivity. By this reasoning, northern forests could act as stronger carbon sinks, partially offsetting greenhouse gas emissions. This expectation assumes that historically positive growth–temperature relationships will continue under warming^1–3^. However, warming not only alleviates cold limitation but also intensifies atmospheric demand for water, increasing vapor pressure deficit and reducing carbon uptake through stomatal regulation. This raises the possibility that water availability may replace temperature as the dominant control on forest growth. Warming may extend the growing season in spring, but it can also accelerate the onset and severity of summer drought stress, so that the period of optimal growth is shifted earlier in the year rather than being lengthened overall^4–6^.

Because warming alters multiple aspects of climate simultaneously, the dominant constraints on growth may also shift through time. For example, Girardin et al.^7^, D’Orangeville, et al.^8^ and Babst, et al.^9^ showed that growth initially increases with warming but later declines once higher temperatures coincide with low moisture availability. This breakdown of the positive growth–temperature relationship, known as the “divergence problem,” has been widely reported across northern ecosystems^10^. These studies suggest that climate limitations on tree growth are not fixed properties of ecosystems but dynamic states that shift with background climate. Once a forest becomes limited by a new factor, physiological or structural adjustments may reinforce that state even if climate conditions later revert^11^. Furthermore, such limitations vary geographically and ecologically, reflecting differences in species’ adaptive capacity and local climate regimes^6,11,12^.

The Canadian Forest Service tree-ring database CFS-TRenD^13^; provides an unprecedented resource to evaluate these dynamics at subcontinental scale, containing more than 40,000 chronologies from over 4000 sites across Canada’s boreal and temperate forests. Dendroclimatology is well suited for this task, as tree-ring chronologies generally track forest productivity well^14^, and provide long-term records that capture changes in climatic limitation through time. Here, we analyze 120 years of growth–climate relationships across Canada to classify forest responses. We ask how these limitation regimes are distributed across regions, how they persist or shift through time, and whether their dynamics are consistent with ecological hypotheses of threshold behavior, state-dependence, and adaptive variability.

Our analysis is structured around three broad questions that reflect threshold, state-dependence, and niche-related variability in forest responses to climate. First, we quantify the prevalence of cold- versus drought-limited growth across Canada’s forests. Based on prior evidence, cold limitation is expected mainly at high latitudes and elevations, while drought limitation should dominate in western and southern boreal regions. This pattern would be consistent with a threshold hypothesis, in which forests cross from cold to drought limitation once long-term climatic moisture balance declines beyond a critical point^15^. Second, we evaluate whether limitation regimes persist or change through time. In line with the state-dependence hypothesis, present-day growth responses may reflect the influence of past climatic conditions, with forests showing plastic adjustments that track recent climate history rather than shifting instantaneously to new limiting factors^10^. Third, we consider whether the stability of growth responses varies among species and regions. Consistent with the lability–niche breadth hypothesis, forests dominated by ecological generalists with broad climatic ranges may exhibit multiple response types, whereas those composed of climatic specialists are expected to show greater consistency in limitation type^11,12^.

Together, these lines of inquiry allow us to interpret spatial and temporal patterns of growth limitation in terms of the ecological processes that govern forest resilience and reorganization under climate change, and to assess whether expectations of enhanced carbon sequestration in northern ecosystems are realistic.

## Results

### Climatic response types by region

To identify broad modes of climatic control on tree growth, we analyzed correlations between annual ring-width indices and monthly temperature and precipitation. The analysis was carried out for 14,101 chronologies that represented aggregated site × species × period combinations (a breakdown of sample sizes is provided in Supplementary Table 3). Chronologies with similar seasonal correlation patterns were grouped into seven response types, ranging from strongly drought-limited to strongly cold-limited. These response types describe growth-climate relationships at the site level and are used throughout the manuscript to characterize patterns of climatic limitation. These groups, hereafter referred to as clusters 1–7, capture the principal axes of forest growth responses to temperature and moisture across Canada.

**Fig. 1:**
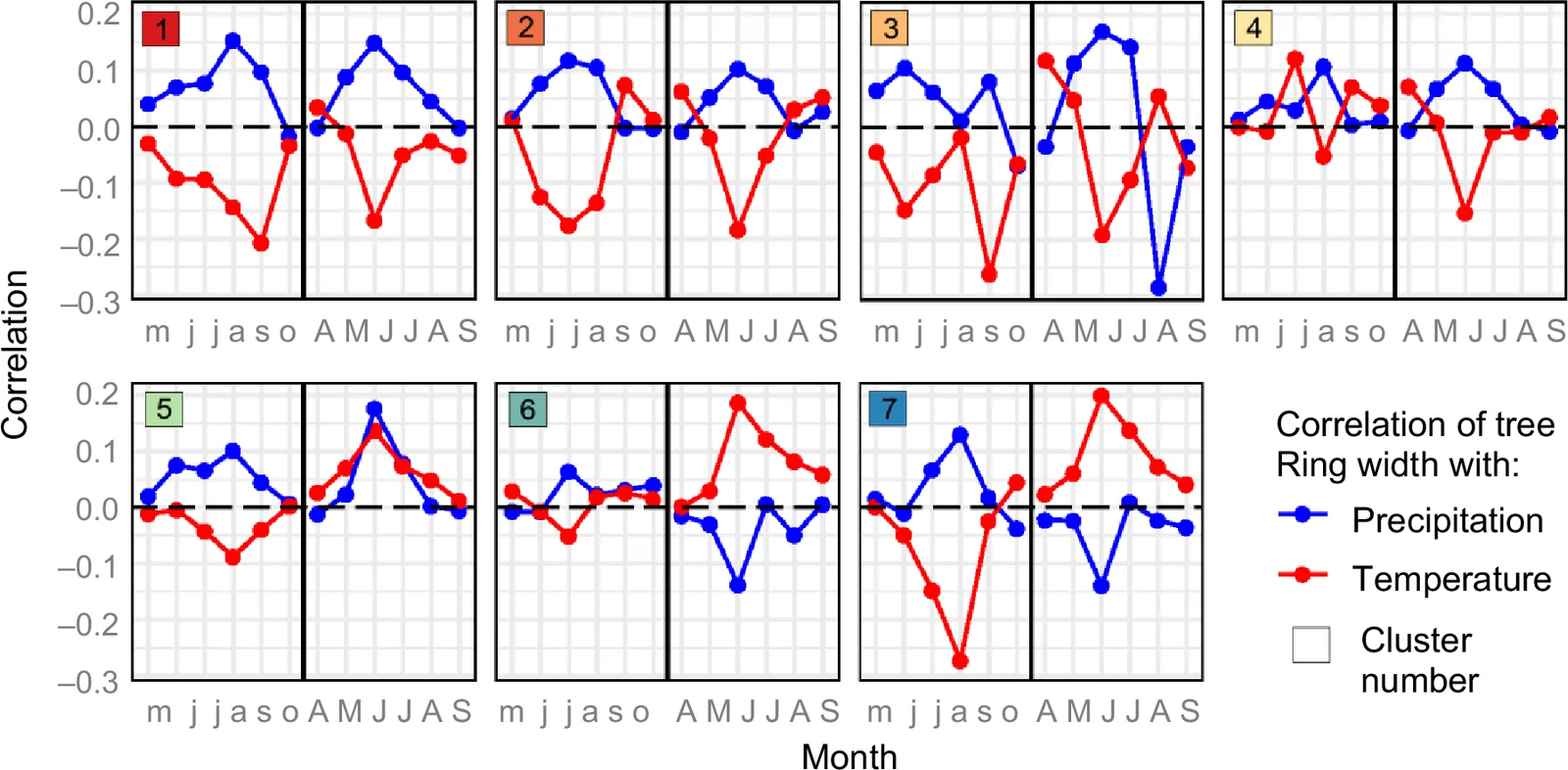
Climate sensitivity types of tree-ring chronologies. Chronologies are grouped by similarity in climate response with multivariate recursive partitioning. Plots show the average correlations of seven chronology groups, as Pearson correlation coefficient between current year’s standardized ring width and monthly temperature and precipitation. Lower-case letters represent climate during the previous year’s growing season, and upper-case letters the current growing season from April to September. Monthly climate variables during the omitted dormancy period (vertical line) do not show strong correlations with tree-ring width (data not shown). Due to the large sample size, the standard errors for corresponding monthly correlation are too low to be visualized (from 0.002 to 0.01).

**Fig. 2:**
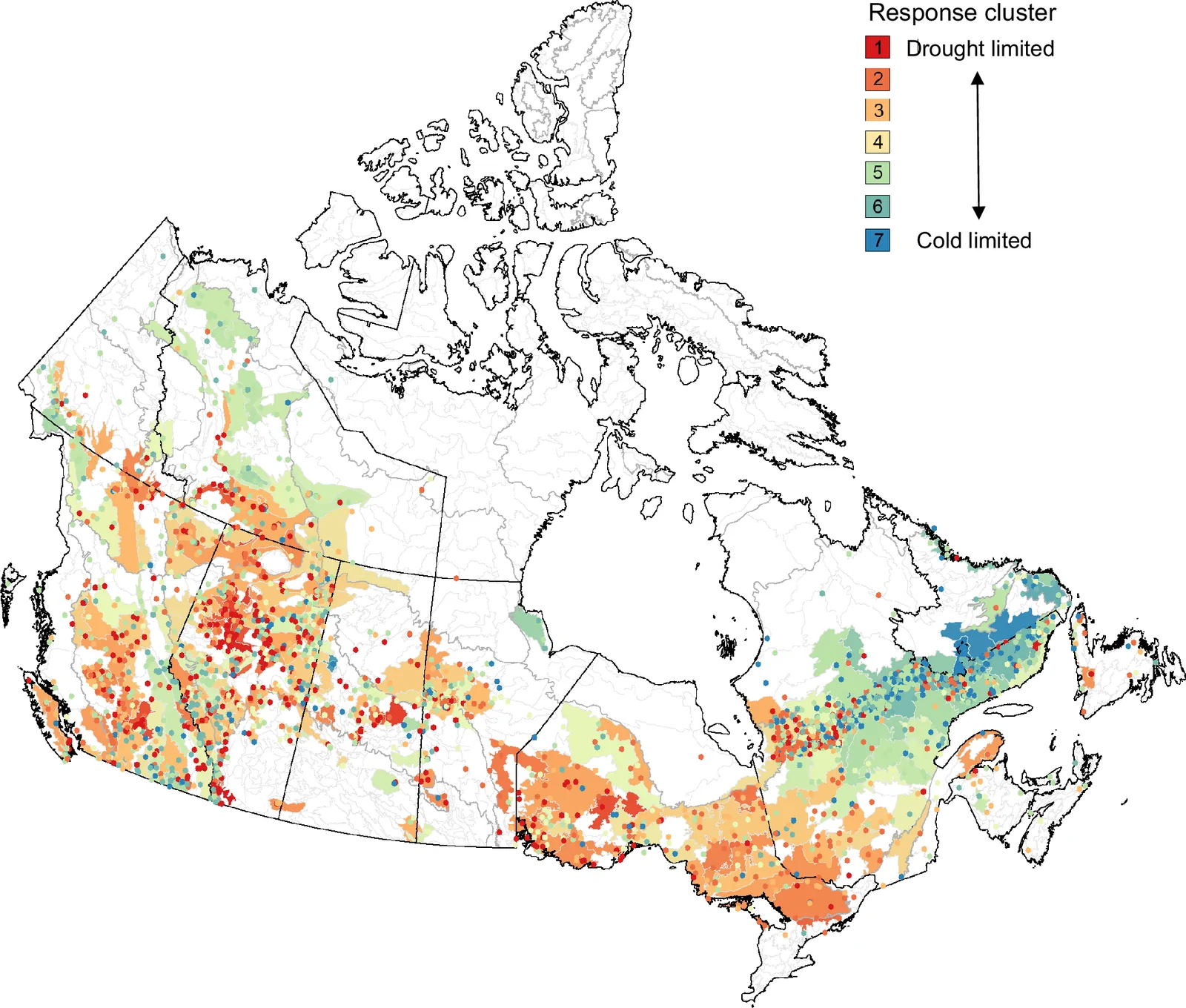
Map of climate sensitivity types aggregated by ecodistricts of Canada. Climate sensitivity types (as shown in Fig. 1) color coded from drought limited (red shades) to cold limited (blue shades). Because points for chronology sites overlap in this map, a color representing the average cluster number was also applied to each ecodistrict, where data was available from at least 10 chronologies. A breakdown of response types by three consecutive 40-year periods is provided as Supplementary Fig. 1.

For the second half of the 20th century where data coverage was best (1951–2000), drought-limited responses (Fig. 1, clusters 1–4) were characterized by negative correlations with temperature and positive correlations with precipitation, differing mainly in the seasonal timing of sensitivity. These responses dominated southern and interior regions, particularly the Prairie–forest transition, central Alberta, southern Ontario and Quebec, and the interior plateaus of British Columbia (Fig. 2). Cold-limited responses (clusters 5–7) showed the opposite pattern, with positive correlations with temperature and weak or negative responses to precipitation. Cluster 5 combined mild cold and drought limitation, while clusters 6 and 7 represented purely cold-limited regimes, the latter often showing reduced growth following an unusually warm prior growing season. These cold-limited responses predominated at high latitudes, in the eastern boreal region, and along mountain ranges of western Canada (Fig. 2).

When aggregated by ecodistrict, drought-limited forests occupied roughly 1.6 million km², or nearly half of Canada’s total forested area, while cold-limited types covered about 0.34 million km², representing one tenth of the total. The remaining 1.5 million km² lacked sufficient chronology density (fewer than ten sites per ecodistrict) to assign a response type. For further details refer to Supplementary Table 1.

### Climatic response types by species

**Fig. 3:**
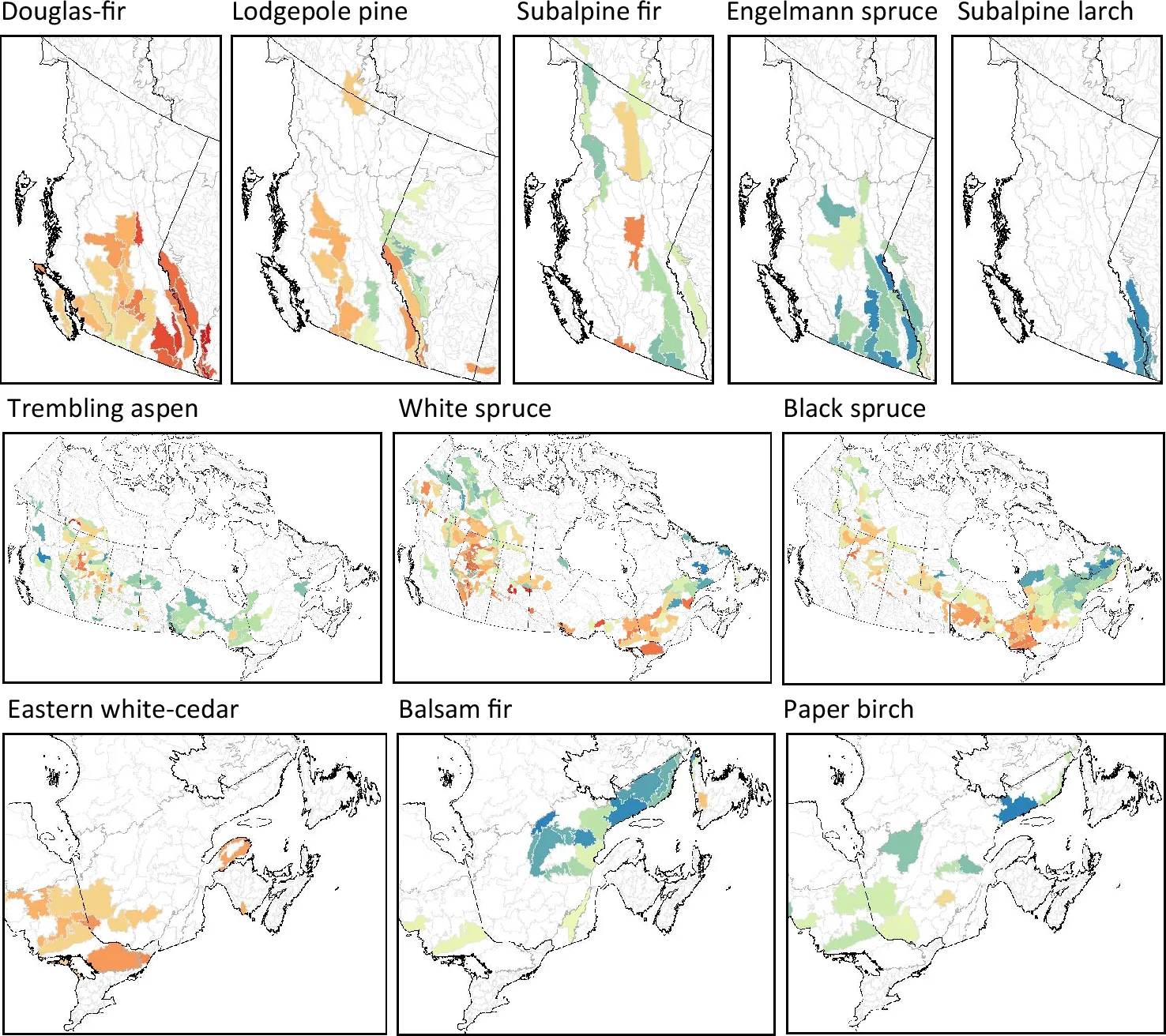
Map of climate sensitivity types by species and ecodistricts. Climate sensitivity types (as shown in Figs. 1 and 2) broken down by species and color coded from drought limited (red shades) to cold limited (blue shades) for ecodistricts with at least 4 chronology sites per species. Color coding as in Figs.1 and 2.

Patterns of climatic limitations varied among species in ways consistent with the lability–niche breadth hypothesis. Species confined to dry, continental environments were largely drought-limited, while those restricted to cold, high-latitude, or montane environments were primarily cold-limited (Table 1, Fig. 3). In contrast, widespread boreal species expressed a broader range of response types, spanning both drought and temperature limitation depending on local conditions. For example, black spruce and white spruce showed a pronounced latitudinal gradient, shifting from predominantly drought-limited responses in the southern boreal to cold-limited responses at northern latitudes. Other wide-ranging species, such as trembling aspen, also showed strong regional contrasts in response types.

**Table 1:** Frequency of climatic sensitivity types 1 through 7, as in Fig. 1, in units of percent of chronologies within each species

		Climate sensitivity type							
Species	Code	1	2	3	4	5	6	7	N
Ponderosa pine	PINUPON	59	3	21	14	3	0	0	29
Douglas fir	PSEUMEN	18	46	12	16	7	1	1	147
Lodgepole pine	PINUCON	30	26	5	9	13	11	5	543
White spruce	PICEGLA	18	36	2	12	7	6	18	214
Jack pine	PINUBAN	28	11	8	21	18	8	5	391
Trembling aspen	POPUTRE	17	26	10	9	17	14	7	1518
Black spruce	PICEMAR	30	12	5	11	26	12	4	561
Tamarack	LARILAR	19	20	0	13	29	15	4	112
Paper birch	BETUPAP	16	25	1	4	7	29	16	68
Balsam fir	ABIEBAL	13	18	4	11	24	20	11	84
Subalpine fir	ABIELAS	9	21	1	15	14	26	14	118
Engelmann spruce	PICEENG	7	10	3	13	12	15	40	86
Subalpine larch	LARILYA	0	0	0	0	21	42	37	19
*N* (incl. other)		942	1008	291	538	722	568	318	4415

Species are sorted from primarily occurring under drought limited conditions (type 1) to cold limited conditions (type 7).

Overall, forests dominated by climatic specialists tended to occupy a narrow, consistent response type, whereas generalist species with broad geographic and climatic ranges were represented across a wide spectrum of response clusters. A breakdown of species-level frequencies by response types is provided in Table 1, which conforms to the expectation that the most specialized taxa showed the least variability in climatic limitations. The more notable result is that the dominant boreal and temperate forest species, which are not specialized for extreme environments, exhibited a remarkably even and continuous distribution of response types. This wide spread of growth–climate relationships among generalist species may indicate greater genetic or physiological adaptive capacity across their geographic ranges.

### Modes of forest response to climate change

**Fig. 4:**
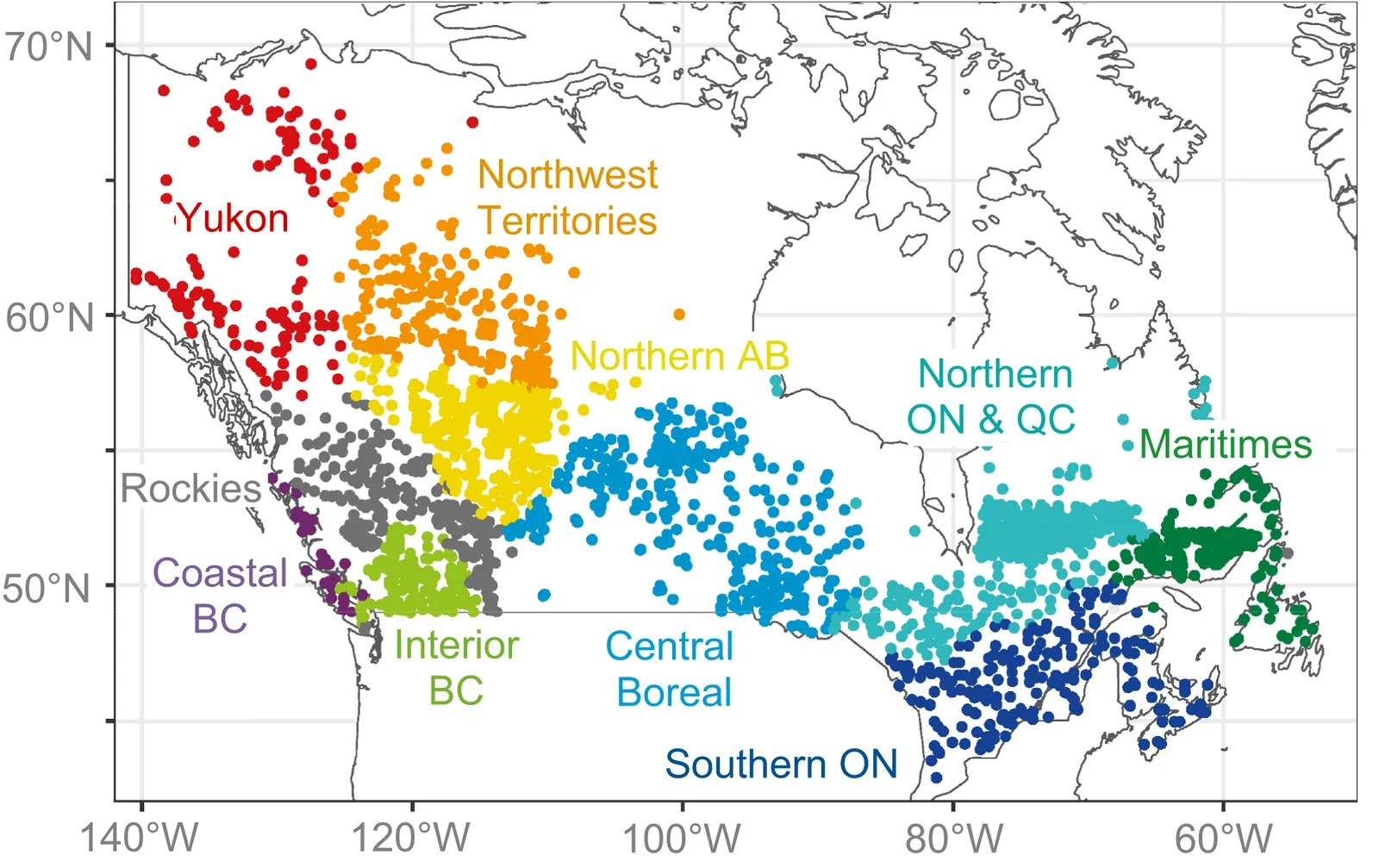
Map of regions with similar climate change trajectories over the last 120 years. Climate change regions were derived by clustering chronologies by similarity of changes from 1901 to 2020 in three climate variables: mean annual temperature, mean annual precipitation, and climate moisture index.

To examine how growth–climate relationships evolved through time, we grouped chronology sites into ten climate-change regions representing coherent trajectories of temperature and moisture change (see Methods). These climate clusters are used as regional groupings (Fig. 4) to summarize shared climate histories and provide context for interpreting temporal shifts in response types, rather than to predict response types directly. These regions were identified by clustering sites according to standardized decadal anomalies in mean annual temperature, precipitation, and climatic moisture index, ensuring that each group reflects a distinct climatic history.

Across these regions, climatic trajectories over the past century showed consistent warming but contrasting moisture trends. Mean annual temperature increased everywhere by roughly +0.5 to +1.0 °C between the mid- and late 20th century, with the strongest warming in northern Alberta and the Northwest Territories groups. Precipitation patterns were less uniform: mean annual and growing-season precipitation rose by 3–9% in western and central Canada, but gains were weaker or absent in northern Ontario, Quebec, and the Maritimes.

When temperature and precipitation are considered together, a two-phase trajectory emerges. From the early to mid-20th century, most regions experienced improved water balance as rising precipitation offset moderate warming. After 1980, this trend slowed or reversed. Western and central regions retained modest moisture gains, whereas northern Alberta, the Northwest Territories, and eastern Canada developed increasing climatic moisture deficits as warming outpaced precipitation. These regional climate histories provide the context for the ecological response modes described below, with a detailed breakdown tracking multiple relevant climate variables available as Supplementary Table 2.

In Interior British Columbia, Douglas-fir (PSEUMEN) and lodgepole pine (PINUCON) were generally the most drought-limited species in the dataset. Response type values showed only minor, statistically non-significant changes across periods, indicating that in the context of consistent warming trends combined with increases in precipitation, their drought sensitivity remained unchanged (Table 2).

**Table 2:** Changes by average climatic sensitivity type (as in Fig. 1) in three consecutive 40-year periods

Region	Response type			Sample size			P-values			Chronology age			Age difference	
Species code	P1	P2	P3	P1	P2	P3	Δ12	Δ23		P1	P2	P3	Δ12	Δ23
Interior BC														
PINUCON	3.2 ( ± 0.2)	2.7 ( ± 0.2)		37	44		0.206			89	125		36	
PSEUMEN	3.0 ( ± 0.1)	3.1 ( ± 0.1)	2.2 ( ± 0.2)	118	136	53	0.356	0.033		63	95	129	33	34
Northwest Territories														
PICEGLA	4.6 ( ± 0.1)	3.0 ( ± 0.1)	2.7 ( ± 0.1)	150	184	120	<0.001	0.029		49	87	125	38	38
PICEMAR	4.8 ( ± 0.2)	3.3 ( ± 0.1)	3.1 ( ± 0.2)	75	103	75	<0.001	0.367		39	78	118	39	41
PINUBAN	5.1 ( ± 0.2)	3.3 ( ± 0.2)	3.1 ( ± 0.2)	79	95	80	<0.001	0.105		56	95	135	39	40
POPUTRE	4.7 ( ± 0.2)	4.4 ( ± 0.1)	2.4 ( ± 0.1)	54	124	112	0.597	<0.001		53	90	130	37	40
Northern AB														
PICEGLA	4.1 ( ± 0.2)	2.7 ( ± 0.1)	3.0 ( ± 0.1)	56	112	101	<0.001	0.338		9	46	86	37	40
PICEMAR	4.1 ( ± 0.3)	3.4 ( ± 0.2)	3.3 ( ± 0.2)	25	52	49	0.402	0.921		24	60	100	36	40
POPUTRE	3.9 ( ± 0.3)	3.3 ( ± 0.1)	3.3 ( ± 0.1)	30	188	187	<0.001	0.946		30	66	106	37	39
Central Boreal														
PICEGLA	2.9 ( ± 0.3)	2.7 ( ± 0.2)	2.8 ( ± 0.3)	24	45	29	0.495	0.787		17	55	94	38	40
PICEMAR	3.8 ( ± 0.1)	2.8 ( ± 0.1)	4.1 ( ± 0.1)	360	630	582	<0.001	<0.001		15	54	94	39	40
PINUBAN	4.3 ( ± 0.3)	2.8 ( ± 0.1)	3.6 ( ± 0.1)	31	126	96	<0.001	0.004		26	61	101	35	40
POPUTRE	4.2 ( ± 0.3)	3.6 ( ± 0.1)	4.5 ( ± 0.1)	28	150	128	0.15	0.001		17	53	94	36	40
Northern ON & QC														
PICEGLA	4.1 ( ± 0.3)	4.0 ( ± 0.3)		24	25		0.697			122	161	198	39	37
PICEMAR	6.0 ( ± 0.1)	3.9 ( ± 0.1)	4.8 ( ± 0.1)	420	530	476	<0.001	<0.001		86	125	165	39	40
PINUBAN	4.9 ( ± 0.2)	3.8 ( ± 0.1)	5.5 ( ± 0.1)	51	121	101	0.002	<0.001		74	114	154	39	40
Maritimes														
PICEMAR	5.6 ( ± 0.1)	5.2 ( ± 0.1)	5.2 ( ± 0.1)	183	205	172	0.096	0.987		90	129	170	40	41

The periods are P1 (1901–1940), P2 (1941–1980), P3 (1981–2020). To be included in comparisons, a minimum sample size of 20 per region was required. Smaller numbers indicate more drought-limited response types. Average chronology ages and age differences among periods are also given to exclude the possibility that observed changes in response types could be explained by age effects. See Table 1 for common names of tree species codes. Supplementary Fig. 1 provides a map summary of of response types by periods.

The Northwest Territories and northern Alberta groups both showed clear transitions from cold- to drought-limited growth, reflecting regional warming that outweighed only modest gains in water balance. In both regions, species shifted from moderately cold-limited responses early in the century to more drought-limited responses in the second and third period. White spruce (PICEGLA), black spruce (PICEMAR), jack pine (PINUBAN), and trembling aspen (POPUTRE) all exhibited this trend, with aspen showing the largest late-century decline in response type values. The two regions differed slightly in trajectory: the Northwest Territories saw only weak mid-century improvement in water balance, whereas northern Alberta experienced a stronger, though short-lived, wetting phase before reverting to renewed drought limitation after 1980.

In the Central Boreal as well as Northern Ontario & Quebec, species showed mid-century shifts toward stronger drought limitation, followed by late-century reversals toward more cold-limited responses (Table 2). Black spruce, jack pine, and trembling aspen all became more drought-limited between the first and second periods but shifted back toward cold limitation by the third. White spruce remained more drought-limited than other species across all periods. These changes coincided with steady increases in temperature and precipitation. However, they appear to be decoupled from changes in water balance, as indicated by dryness indices, which somewhat improved from the first to second period but remained neutral or reversed in the second period.

In the Maritimes, black spruce remained consistently cold-limited across all three periods, with only minor variation in response type values (Table 2). Climatic trends showed warming across both transitions, while precipitation gains from the first to second period were followed by declines after 1980. This produced neutral to negative changes in water balance indices in the late century. Despite these drier conditions, growth responses of black spruce did not shift from their cold-limited state.

## Discussion

### Most Canadian forests are already drought limited, not cold limited

Our analysis shows that Canadian forests are more often constrained by water availability than by low temperatures. For the 1951–2000 period, drought-limited response types accounted for about 47% of the sampled forest area, while cold-limited types represented only around 10% (Fig. 2, Supplementary Table 1). This finding challenges the traditional assumption that the productivity of northern forests is primarily restricted by cold and short growing seasons. Our results conform to prior research in Canada, showing that drought stress already constrains the Canadian boreal forest, particularly in its western and southern range^5,16–18^. Water limitations already dominate across much of the continental interior and southern boreal.

A key caveat of our analysis is that nearly 43% of Canada’s forest area could not be classified due to insufficient chronology density. These under-sampled areas include coastal British Columbia and the far northern boreal/forest–tundra transition. Both zones are more likely to harbor cold-limited growth responses, given their cool, moist climates and short growing seasons. If these regions were better represented, the proportion of cold-limited forests at the national scale would likely increase. With the exception of coastal BC forests, the chronology network captures the regions where the largest portion of Canada’s commercially valuable and accessible forest resources are concentrated, namely the more southern boreal and sub-boreal forest regions. In contrast, the northern cold-limited forests often consist of sparse, slow-growing stands with low biomass accumulation. These forests could contribute to carbon sequestration in the long-term future, but this inference is not supported by data analyzed in this study.

Taken together, the data indicates that while cold-limited forests may be common in under-sampled regions, the practical picture for Canada’s managed or productive forests is one of widespread drought limitation. This aligns with other research, concluding that warming-induced drought limitations are emerging as the dominant constraint on Canadian forest growth and regeneration^5,17,19^. Consequently, the expectation of broad productivity gains under climate warming may not be warranted for much of the forest land that matters for management and carbon budgets.

Although not specifically modeled in this study, the finding of widespread water limitations has potential implications for projections of future forest productivity. Without substantial increases in precipitation, further warming will intensify evapotranspirative stress, potentially pushing already moisture-limited systems into deeper drought sensitivity. This behavior would reflect threshold dynamics, such as those described at the boreal–parkland transition zone^15^. However, our results suggest these mechanisms are evident in some regions across boreal and sub-boreal Canada. Thus, productivity gains from warming may be offset, or even reversed, by increased drought stress.

### Four characteristic modes of growth response to climate change

Our results point to multiple modes of behavior. In some regions, warming has pushed forests across moisture thresholds into persistent drought limitation. In others, growth responses have remained largely constant over time. In a third set of regions, growth limitations have reversed, reflecting plastic adjustments that decouple growth sensitivity from broad climate trends. Finally, in regions with high baseline precipitation and excess soil moisture, cold limitation remains stable even under warming.

In Interior British Columbia, Douglas-fir and lodgepole pine are among the most drought-limited populations but showed little change across periods. Here, warming and precipitation gains appear to have roughly balanced, maintaining a stable drought-sensitive state. However, these species already operate close to their physiological thresholds at the forest-grassland transition. Any further increase in evaporative demand without a corresponding increase in precipitation could tip them into more severe drought stress.

In the Northwest Territories and northern Alberta, late-century warming outweighed only modest gains in water balance, and species shifted from moderately cold-limited to consistently drought-limited growth. This pattern is expected when forests cross critical moisture thresholds^15^ and is consistent with observed drought-related growth reductions and mortality in western Canada over recent decades^17,18,20,21^. Continued warming in these regions may increase the risk of further drought limitation if precipitation or soil water availability does not increase.

In contrast, forests of the central Boreal and northern Ontario and Quebec showed mid-century shifts toward stronger drought limitation followed by late-century reversals back toward cold-limited responses (Table 2). The reversible shifts likely reflect physiological acclimation rather than direct tracking of climate variables. Trees can adjust leaf area, stomatal regulation, carbon allocation, and aspects of hydraulic architecture in response to repeated drought exposure, thereby modifying the balance between temperature and water limitations on growth^22^. Reductions in leaf area or stomatal conductance can lower transpirational demand, while longer-term adjustments in water-transport capacity and allocation may increase hydraulic safety under dry conditions. In addition, such responses may persist beyond the drought event itself as legacy effects, contributing to delayed or reversible changes in growth–climate relationships even under continued warming^23–25^. Taken together, these patterns suggest a reversible mode of growth–climate relationships, where stands can oscillate between drought and cold limitation depending on the climate history and plastic adjustments. Therefore, growth–climate relationships documented in the past do not necessarily predict future trajectories with certainty.

Finally, in the Maritimes, black spruce, a dominant species on wet sites, remained consistently cold-limited across all periods (Table 2), despite warming and late-century declines in water balance (Supplementary Table 2). The high baseline precipitation in this region appears sufficient to buffer against drought sensitivity, keeping growth constrained more by temperature and growing season length than by water availability. This provides a counterpoint to the more drought-sensitive continental interior, highlighting how baseline hydroclimate conditions, e.g., excess soil moisture^26^, mediate whether warming translates into drought or cold limitation.

### Species and population-specific response types

Species-specific differences reveal contrasts between ecological generalists and specialists. Trembling aspen and jack pine, for instance, exhibited flexible growth responses across their ranges, switching between drought and cold limitation depending on local conditions. This flexibility likely reflects broad ecological tolerances and high physiological plasticity. In contrast, species such as Engelmann spruce and subalpine fir adapted to cold, high-elevation environments, consistently exhibited cold-limited responses, suggesting narrower niche specialization. Black spruce provides an intermediate case, with cold-limited responses in northern and eastern ranges but drought sensitivity in more southerly regions^5^. We should note, however, that regional variation in response types of widespread species may reflect interacting influences of macroclimate, site conditions, and population-level genetic variation, which cannot be disentangled with observational data alone. For example, black spruce commonly occurs on wet or poorly drained sites associated with topographic convergence or soil conditions that promote waterlogging. If such topo-edaphic factors buffer drought responses more strongly in regions such as the Maritimes than elsewhere, they could contribute to the more persistent cold-limited behavior observed there. However, these influences cannot be separated from regional climatic differences, except through experimental research.

High-elevation species with cold-limited response types, namely Engelmann spruce and subalpine larch, showed a unique response pattern: opposite correlations with temperature in the current versus previous growing season (Fig. 1, Cluster 7). Warm temperatures in the previous growing season had a negative carry-over effect on ring growth, whereas warmth during the current growing season had a positive effect on the annual growth increment. This lagged effect may reflect carbohydrate depletion: when warm late summers promote extensive latewood formation, trees may exhaust carbon reserves, leaving insufficient resources for earlywood production the following spring. Conversely, cooler, shorter seasons may promote carbon storage, supporting stronger early-season growth in the subsequent year. These lagged effects underscore the importance of non-structural carbohydrate allocation in cold-limited systems.

From a management perspective, this diversity of responses has practical implications. Species with flexible responses, such as most widespread boreal species (Table 1, middle section), may remain adaptable to climate change, except for the already drought-limited populations at their range margins. Conversely, niche specialists with unique response types, such as Engelmann spruce and subalpine larch, may shift more rapidly outside their climate niche. In practice, assisted migration is therefore most compelling in drought-prone western boreal regions, where local seed sources already operate near physiological thresholds. High-elevation and niche-specialist species in interior BC and the Rockies also warrant forward-looking seed transfers, while in the central and eastern boreal forest conventional seed sourcing may remain viable for now. That said, these conclusions are based on changes to limiting climatic factors, not absolute growth, since our tree-ring indices were standardized. As a result, shifts in growth limitation do not necessarily translate into corresponding changes in absolute productivity, but rather reflect changes in the relative importance of climatic constraints on growth. This means that even if limiting factors remain unchanged, assisted migration could still prove valuable. Provenance trials and common garden experiments have consistently shown that southern provenances often outperform local seed sources under recent decades of warming.

## Conclusions

This study shows that Canadian forests are not poised to experience consistent productivity gains under continued climate warming. While northern forests are often assumed to be limited by cold, our analysis demonstrates that drought sensitivity already dominates much of the boreal and sub-boreal domain. Where warming has pushed systems across moisture thresholds, such as in northern Alberta and the Northwest Territories, forests have shifted toward drought-limited states. In contrast, regions such as the Central Boreal and northern Ontario and Quebec reveal more reversible growth–climate relationships, with species oscillating between drought and cold limitation depending on recent climate history. Interior British Columbia remains drought-limited but relatively stable, while the Maritimes continue to be constrained by cold, buffered by high baseline precipitation.

The broader implication is that Canada’s forests may not serve as a carbon sink that will necessarily strengthen with warming. Instead, their role in climate mitigation will depend on a shifting balance between regions that lose productivity to drought and others that retain or regain cold limitation. For reforestation and regeneration programs, this means tailoring strategies to regional climate trajectories and species-specific sensitivities. In drought-prone regions such as northern Alberta and the Northwest Territories, “business as usual” planting of local seed sources will likely reinforce vulnerability. Here, prioritizing alternative species choices or drought-tolerant genotypes should help maintain forest cover and productivity. In contrast, in the central and eastern boreal forest regions, where growth–climate responses remain stable or have shown reversible patterns, natural regeneration or conventional reforestation practices may remain viable for some time. In short, climate change adaptation in reforestation will not be a single national prescription but a regionally differentiated strategy, balancing the urgency of assisted migration in some areas with the continued viability of current management practices in others.

## Methods

### Tree-ring data

This study is based on the Canadian Forest Service tree-ring database CFS-TRenD^13^, which tracks growth of commercially important forest tree species, with more than 80% of the samples representing inventory records. The database contains tree-ring records from 4179 sites across Canada. For this study, we focused on the species with the most chronology sites, which also correspond to Canada’s most common forest trees: black spruce (*Picea mariana* (Mill.) Britton, Sterns & Poggenburg, *n* = 1552 sites), trembling aspen (*Populus tremuloides* Michx., *n* = 536), white spruce (*Picea glauca* (Moench) Voss, *n* = 517), jack pine (*Pinus banksiana* Lamb., *n* = 376), lodgepole pine (*Pinus contorta* Douglas, *n* = 210), Douglas-fir (*Pseudotsuga menziesii* (Mirbel) Franco, *n* = 135), balsam fir (*Abies balsamea* (L.) Mill., *n* = 111), and subalpine fir (*Abies lasiocarpa* (Hook.) Nutt., *n* = 69). Other common Canadian tree species, such as tamarack, paper birch, and sugar maple were excluded due to insufficient sampling density.

We analyzed changes in limiting climate factors both over time and across different ecoregions of Canada. Because the temporal coverage of the CFS-TRenD database is best for the second half of the 20th century, we chose the 1951–2000 period for the spatial analysis. To quantify changes in climate–growth relationships over time, we used three consecutive 40-year periods: 1901–1940, 1941–1980, and 1981–2020. A chronology was included if it had ≥70% data coverage in either the 1901–1940 and 1941–1980 periods, or in the 1941–1980 and 1981–2020 periods. The number of site–species combinations for different periods is provided in Supplementary Table 3.

The pairwise-complete comparison approach controls for changes in chronology composition across periods, allowing consistent comparisons among species, regions, and time slices, although not for confounding with tree age. This is a potential concern, because limiting climate factors have been shown to vary by age class^27^. For our comparisons, this means inferences can only be drawn from the difference observed in one 80-year period (1901–1940 versus 1941–1980) compared to the subsequent 80-year period (1941–1980 versus 1981–2020), where the age effects are similar. Similarly, comparisons of species by region within the same 80-year period, as well as regional comparisons using the same species can be made, with the age effect held constant. Further, we provide a tabulation of average chronology ages among the three periods to investigate the possibility that different response types among species and regions could be caused by age effects. Chronology age increments are consistent among regions and close to the 40-year time steps as intended by the sampling design. They could therefore not explain regional differences or species differences in response types (Table 2).

To further evaluate potential age-related confounding, we repeated the cumulative link mixed model analyses including chronology age as a covariate. This did not materially change the estimated response-type trajectories across periods, species, or regions. We therefore retained the original model specification. Including age as a covariate does not resolve the confounding between age and time because chronology age increases systematically between consecutive periods. In such cases, regression adjustment cannot separate the effects of two correlated variables. Instead, our inference relies on paired comparisons of consecutive 40-year periods where age increments are similar among regions and species, which provides a transparent control for potential age effects.

### Data processing and constructing chronologies

We aggregated 114,798 core measurements into 38,395 tree-level chronologies. Multiple cores from the same tree (replicates, re-samples, or multiple radii) were averaged using least-squares means to account for missing values. Site chronologies were then constructed by combining tree-level chronologies. Separating these steps ensured that quality statistics were not inflated by correlated cores from the same tree. Next, raw tree-ring series were detrended and standardized using a smoothing spline (nyrs = 30) and summarized using a robust biweight mean, implemented with the chron() function of the dplR package^28,29^. The same method was applied in the second step to construct site chronologies. The median number of trees per site chronology was six (10th–90th percentile: 3–20). To remove temporal autocorrelation (prewhitening), we applied autoregressive modeling^30^, implemented in dplR for R^31^. To ensure consistency and remove potentially erroneous data, we excluded a small number of individual tree time series with interseries correlation <0.6 (using interseries.cor in dplR). In total, ~4% of chronologies were removed due to low correlation or data inconsistencies^13^. Site-level chronologies were further assessed using mean inter-series correlation (R-bar) and the Expressed Population Signal^32^. Chronologies with EPS ≥ 0.8 were retained (>80% of sites). Sensitivity tests using lower thresholds did not substantially change results.

### Climate change and growth response analysis

Monthly climate data (1901–2020) were obtained from ClimateNA v7.01^33^. ClimateNA provides downscaled, interpolated station-based climate grids based on PRISM method^34^. The software version used to query historical data is available for download from http://ClimateNA.ca. Climate time series were generated for all chronology sites at two temporal resolutions: monthly (1901–2020) to analyze ring-width response, and decadal to analyze regional climate change trends. To summarize climate trends, we grouped sites into 10 “climate-change regions” using k-means clustering^35^ of standardized decadal anomalies (z-score scaling) in mean annual temperature, precipitation, and climatic moisture index^15^, giving 36 variables per site (12 decades × 3 variables). The clustering was performed with the *kmeans()* function in the R *stats* package^31^.

Variation in tree-ring width reflects cumulative effects of climate conditions from the current and previous growing seasons. We evaluated growth–climate associations using lagged correlations between standardized annual ring-width indices and monthly climate variables. We calculated correlations, over 1951–2000 and the three consecutive 40-year periods, from the previous year’s April through the current year’s October, covering two complete growing seasons of potential climate influence. Monthly temperature and precipitation data were detrended with the same 30-year smoothing spline used for tree rings, following best practice to strengthen climate–growth correlations^36^, although in this study, the additional detrending left the results essentially unchanged. Chronologies with similar climate–growth correlation patterns were grouped into response types using multivariate recursive partitioning implemented with the mvpart package^37^. The number of clusters (*n* = 7) was chosen based on a scree plot of residual error, identifying a point beyond which additional clusters provided only marginal reductions in residual variance. Within each group, chronologies shared comparable seasonal correlation structures. Additional clusters did not represent distinct or interpretable response types, but rather subdivided a continuum of growth–climate relationships.

To test whether the distribution of the ordinal response variable (ordered cluster numbers ranging from 1–7) changed between time periods, we fit a cumulative link mixed model (CLMM) with a logit link using the R package ordinal^38^. In these models, the cluster number was treated as an ordered categorical response, period was included as a fixed effect, and site chronology ID was included as a random intercept. For each species × region combination, separate models were fit to compare the first two periods (1901–1940 vs. 1941–1980) and the last two periods (1941–1980 vs. 1981–2020). Significance of period effects was assessed using a two-sided Wald *z*-tests, and p-values were adjusted for multiple comparisons across all tests using Holm’s correction implemented with the p.adjust function^31,39^.

For visualization and context, we aggregated statistics from this analysis and forest cover grid cells from ESRI 10 m Land Cover data^40^ by ecodistrict of the Canada Terrestrial Ecozones, Ecoregions, and Ecodistricts dataset developed by Agriculture and Agri-Food Canada^41^.

## Supplementary information

Transparent Peer Review file

Supplementary Figs. and Tables

## Data Availability

The raw chronology data is available from the Federal Government of Canada from the repository https://treesource.rncan.gc.ca/en/treerings. Data underlying the figures, tables, and statistical analysis of this manuscript is provided through the repository Figshare at https://doi.org/10.6084/m9.figshare.32441463.

## Statement regarding map creation and data

All maps of this manuscript (Figs. 2–4) and the supplementary material (Fig. S1) were created by the authors with open source GIS software QGIS v3.40.9 (https://qgis.org) and open access GIS data from Natural Earth (https://www.naturalearthdata.com) and public Government of Canada data (https://sis.agr.gc.ca/cansis/nsdb/ecostrat/gis_data.html).
