# Supplementary material for: Shifting growth–climate limitations in Canadian forests under recent climate change
Source: Commun Earth Environ. 2026 Jun 26;7(1):828. doi: 10.1038/s43247-026-03713-7 (PMC13672052; doi:10.1038/s43247-026-03713-7)
Supplement: Supplementary file 1 [file 43247_2026_3713_MOESM1_ESM.pdf]

nature portfolio

Peer Review File

Shifting growth-climate limitations in Canadian forests under recent climate change

Corresponding Author: Dr Zihaohan Sang

This file contains all editorial decision letters in order by version, followed by all author rebuttals in order by version.

Version 0:

Decision Letter:

** Please ensure you delete the link to your author home page in this e-mail if you wish to forward it to your coauthors **

Dear Dr Sang,

Your manuscript titled "Drought Sensitivity and Diverse Growth Responses of Canadian Forests to Climate Warming" has now been seen by 3 reviewers, and we include their comments at the end of this message. They find your work of interest, but some important points are raised. We are interested in the possibility of publishing your study in Communications Earth & Environment, but would like to consider your responses to these concerns and assess a revised manuscript before we make a final decision on publication. In revision, please fully consider potential factors influencing the tree climate sensitivity, including tree age as suggested by reviewer #2.

We therefore invite you to revise and resubmit your manuscript, along with a point-by-point response that takes into account the points raised. Please highlight all changes in the manuscript text file.

Please submit your point-by-point responses as a separate file, distinct from your cover letter where you can add responses to the Editors' comments that you do not want to be made available to the reviewers. Word files are preferred. We recommend that any figures, tables or graphs that are included in the response to reviewers are also included in the main article or Supplementary Information.

We are committed to providing a fair and constructive peer-review process. Please don't hesitate to contact us if you wish to discuss the revision in more detail.

Please use the following link to submit your revised manuscript, point-by-point response to the referees' comments (which should be in a separate document to any cover letter), a tracked-changes version of the manuscript (as a PDF file) and the completed checklist:
Link Redacted
** This url links to your confidential home page and associated information about manuscripts you may have submitted or be reviewing for us. If you wish to forward this email to co-authors, please delete the link to your homepage first **

We hope to receive your revised paper within six weeks; please let us know if you aren't able to submit it within this time so that we can discuss how best to proceed. If we don't hear from you, and the revision process takes significantly longer, we may close your file. In this event, we will still be happy to reconsider your paper at a later date, as long as nothing similar has been accepted for publication at Communications Earth & Environment or published elsewhere in the meantime.

Please do not hesitate to contact us if you have any questions or would like to discuss these revisions further. We look forward to seeing the revised manuscript and thank you for the opportunity to review your work.

Best regards,

Erika Buscardo, PhD
Editorial Board Member
Communications Earth & Environment
orcid.org/0000-0003-4029-8465

Mengjie Wang, PhD
Associate Editor, Communications Earth & Environment
Consulting Editor, Communications Sustainability

EDITORIAL POLICIES AND FORMATTING

We ask that you ensure your manuscript complies with our editorial policies. Please ensure that the following formatting requirements are met, and any checklist relevant to your research is completed and uploaded as a Related Manuscript file type with the revised article.

For Manuscripts that fall into the following fields:
• Behavioural and social science
• Ecological, evolutionary & environmental sciences
• Life sciences
An updated and completed version of our Reporting Summary must be uploaded with the revised manuscript
You can download the form here:
https://www.nature.com/documents/nr-reporting-summary.pdf

Furthermore, please align your manuscript with our format requirements, which are summarized on the following checklist: Communications Earth & Environment formatting checklist

and also in our style and formatting guide Communications Earth & Environment formatting guide .

*** DATA: Communications Earth & Environment endorses the principles of the Enabling FAIR data project (http://www.copdess.org/enabling-fair-data-project/). We ask authors to make the data that support their conclusions available in permanent, publically accessible data repositories. (Please contact the editor if you are unable to make your data available).

All Communications Earth & Environment manuscripts must include a section titled "Data Availability" at the end of the Methods section or main text (if no Methods). More information on this policy, is available at http://www.nature.com/authors/policies/data/data-availability-statements-data-citations.pdf.

In particular, the Data availability statement should include:
- Unique identifiers (such as DOIs and hyperlinks for datasets in public repositories)
- Accession codes where appropriate
- If applicable, a statement regarding data available with restrictions
- If a dataset has a Digital Object Identifier (DOI) as its unique identifier, we strongly encourage including this in the Reference list and citing the dataset in the Data Availability Statement.

DATA SOURCES: All new data associated with the paper should be placed in a persistent repository where they can be freely and enduringly accessed. We recommend submitting the data to discipline-specific, community-recognized repositories, where possible and a list of recommended repositories is provided at http://www.nature.com/sdata/policies/repositories.

If a community resource is unavailable, data can be submitted to generalist repositories such as figshare or Dryad Digital Repository. Please provide a unique identifier for the data (for example a DOI or a permanent URL) in the data availability statement, if possible. If the repository does not provide identifiers, we encourage authors to supply the search terms that will return the data. For data that have been obtained from publically available sources, please provide a URL and the specific data product name in the data availability statement. Data with a DOI should be further cited in the methods reference section.

Please refer to our data policies at http://www.nature.com/authors/policies/availability.html.

REVIEWER COMMENTS:

Reviewer #1 (Remarks to the Author):

This manuscript presents a comprehensive and valuable analysis of tree-growth responses to climate warming across Canadian forests over a 120-year period. The study leverages an extensive tree-ring network to convincingly demonstrate that drought sensitivity, rather than cold limitation, is already the dominant constraint for nearly half of the forested area. The identification of four characteristic temporal response modes (persistent, transitioning, reversible, and buffered) provides a nuanced and important framework for understanding forest dynamics under climate change. The work is data-rich, the

methodology is largely sound, and the conclusions have significant implications for predicting forest productivity and carbon sink potential. The manuscript is suitable for publication after addressing some minor points to improve clarity and precision. I therefore recommend Minor Revisions.

Title: Drought sensitivity was not quantified separately, but rather falls under the broader category of diverse growth responses to climate. It is advised to adjust the title for more precise expression.

The number of sampling sites described in the abstract and the subsequent results section of the paper is inconsistent. Please double-check this.

Line 109:It is recommended to add a cluster analysis of the site chronologies.

The median number of trees included in the site chronologies is 6? Are all the chronologies reliable? Additionally, it is recommended to clarify the reliability of the chronologies across the different time periods analyzed for climate correlation.

The Methods section contains some disorganized and incomplete descriptions of the results. For example, the generation of tree-ring index series, including the detrending methods, should be included in the "Tree-ring data" chapter.

Reviewer #2 (Remarks to the Author):

This manuscript addresses a critical and timely question regarding the dynamic responses of Canadian forests to climate warming, with a specific focus on the shift between cold and drought limitations. By adopting a comprehensive tree-ring dataset from Canada, the research fills a key gap in understanding how boreal forests that have been traditionally assumed to be cold-limited are increasingly constrained by drought. The large spatial and temporal scope provides robust statistical power to detect regional and temporal patterns of growth-climate relationships. The conclusions are reliable. I have the following major concerns for the authors to improve the manuscript:
1. Although the authors mention controlling for age effects by comparing consecutive 40-year periods, tree age can independently modulate climate sensitivity (e.g., younger vs. mature trees). The current analysis assumes age effects are consistent across regions and periods, but this assumption is not explicitly tested. I suggest the authors incorporate tree age as a covariate in the cumulative link mixed models to isolate age-related variation from climate-driven shifts in limitation types. Additionally, provide more detailed information on age distributions within and across chronologies to assess potential confounding.
2. The study identifies reversible shifts between drought and cold limitation in the Central Boreal and Northern Ontario & Quebec regions but offers limited mechanistic explanation for these transitions beyond "plastic canopy and hydraulic adjustments." Integrating trait data (either from literature or existing databases) would strengthen the mechanistic link between climate history, plastic adjustments, and growth responses.
3. While the study contrasts generalist vs. specialist species, some widespread species (e.g., black spruce) show mixed responses across regions. The authors attribute this to local conditions, but it would be valuable to explicitly test whether these regional differences correlate with specific environmental gradients (e.g., aridity, soil texture) or genetic variation.
4. The study uses CMI to characterize water balance, but the relationship between CMI and actual soil moisture availability (which depends on soil properties and topography) is not explicitly validated. In regions with high baseline precipitation (e.g., the Maritimes), soil moisture buffering may decouple CMI from plant-available water. I suggest the authors include a brief discussion of how soil characteristics or topographic variation might modify the relationship between CMI and drought stress, and note this as a potential source of uncertainty in regions with complex terrain.
5. The terms "drought limitation" and "drought sensitivity" are used interchangeably throughout the manuscript, but they are not strictly synonymous (sensitivity refers to responsiveness, while limitation implies a constraint on growth). Suggested revision: Standardize terminology to distinguish between sensitivity (correlation with moisture variables) and limitation (growth reduction due to moisture deficit). This clarification would avoid ambiguity, particularly in discussions of species-specific responses.
Below are some minor concerns:
1.Figure 2 effectively maps spatial patterns of limitation types, but temporal shifts in response types (e.g., cold-to-drought transitions) are only presented in Table 2, which is dense and difficult to interpret. Please add a supplementary figure (e.g., stacked bar charts or heatmaps) showing temporal changes in response type frequencies for key species-region combinations (e.g., white spruce in the Northwest Territories). This would make temporal trends more accessible to readers.
2.The abstract mentions "four characteristic modes of growth response to climate change," but the results and discussion describe five modes (persistent drought, cold-to-drought transition, reversible shifts, stable cold limitation, and the unique lagged response of high-elevation species). Please clarify this discrepancy to avoid confusion.
3.In the Methods section, specify the criteria for selecting the seven response clusters (e.g., the scree plot of residual error should be referenced or included as a supplementary figure to justify the cluster number).
4.The authors note that standardized tree-ring indices were used, which control for growth trends but obscure absolute growth rates. Briefly discuss how this might affect interpretations of productivity changes, as limiting factor shifts do not always translate to absolute growth declines.

Reviewer #3 (Remarks to the Author):

The manuscript by Sang et al., is well written and very interesting. I only have a few minor suggestions for this manuscript.

1. I was a little confused by the seven response clusters of chronologies compared to the ten clusters based on climate trends. I find both of these analyses interesting but was looking for a more in terms of how these two clusters are interacting. The authors talk about this in the text somewhat but I think they could do a better job illustrating how the two clustering schemes interact perhaps in figure 4, which isn't really doing a lot except showing 10 clusters. Can the authors use the 10 climate clusters to help predict the seven response clusters in a model.. perhaps a mixed effect model?

2. It would be cool to see what 40-year period the chronologies switched from cold to drought (for those sites that did) on a map… and then see the ones that reversed back to cold and what 40-year period that happened. Perhaps Table 2 was getting at this but not with correlation values… I was also a bit confused by the gray shading. Having the spatial component of table 1 would be nice to see as well .

** Visit Nature Portfolio's author and referees' website at www.nature.com/authors for information about policies, services and author benefits**

Communications Earth & Environment is committed to improving transparency in authorship. As part of our efforts in this direction, we are now requesting that all authors identified as 'corresponding author' create and link their Open Researcher and Contributor Identifier (ORCID) with their account on the Manuscript Tracking System prior to acceptance. ORCID helps the scientific community achieve unambiguous attribution of all scholarly contributions. You can create and link your ORCID from the home page of the Manuscript Tracking System by clicking on 'Modify my Springer Nature account' and following the instructions in the link below. Please also inform all co-authors that they can add their ORCIDs to their accounts and that they must do so prior to acceptance.
https://www.springernature.com/gp/researchers/orcid/orcid-for-nature-research

For more information please visit http://www.springernature.com/orcid

If you experience problems in linking your ORCID, please contact the Platform Support Helpdesk.

Version 1:

Decision Letter:

** Please ensure you delete the link to your author home page in this e-mail if you wish to forward it to your coauthors **

Dear Dr Sang,

Your manuscript titled "Shifting Growth–Climate Limitations in Canadian Forests under Observed Climate Change" has now been seen by our reviewers, whose comments appear below. In light of their advice we are delighted to say that we are happy, in principle, to publish a suitably revised version in Communications Earth & Environment.

We therefore invite you to revise your paper one last time to address the remaining concerns of our reviewers. At the same time we ask that you edit your manuscript to comply with our format requirements and to maximise the accessibility and therefore the impact of your work.

EDITORIAL REQUESTS:

Please review our specific editorial comments and requests regarding your manuscript in the attached "Editorial Requests Table".

*****Please take care to match our formatting and policy requirements. We will check revised manuscript and return manuscripts that do not comply. Such requests will lead to delays. *****

Please outline your response to each request in the right hand column. Please upload the completed table with your manuscript files as a Related Manuscript file.

If you have any questions or concerns about any of our requests, please do not hesitate to contact me.

SUBMISSION INFORMATION:

In order to accept your paper, we require the files listed at the end of the Editorial Requests Table; the list of required files is also available at https://www.nature.com/documents/commsj-file-checklist.pdf .

OPEN ACCESS:

Communications Earth & Environment is a fully open access journal. Articles are made freely accessible on publication. For further information about article processing charges, open access funding, and advice and support from Nature Portfolio, please visit https://www.nature.com/commsenv/open-access

At acceptance, you will be provided with instructions for completing the open access licence agreement on behalf of all authors. This grants us the necessary permissions to publish your paper. Additionally, you will be asked to declare that all required third party permissions have been obtained, and to provide billing information in order to pay the article-processing charge (APC).

Please use the following link to submit the above items:
Link Redacted
** This url links to your confidential home page and associated information about manuscripts you may have submitted or be reviewing for us. If you wish to forward this email to co-authors, please delete the link to your homepage first **

We hope to hear from you within two weeks; please let us know if you need more time.

Best regards,

Mengjie Wang
Associate Editor, Communications Earth & Environment
Consulting Editor, Communications Sustainability
Bluesky: @commsearth.nature.com; @commssustain.nature.com

Erika Buscardo, PhD
Editorial Board Member
Communications Earth & Environment
orcid.org/0000-0003-4029-8465

REVIEWERS' COMMENTS:

Reviewer #1 (Remarks to the Author):

I noticed that the author has already cleaned the data, retaining more reliable information. The language usage is also more precise, and the discussion is clearer and more in-depth. I think this MS can be accepted. However, there is a minor issue.
A single tree cannot form a chronology. The writing "individual tree chronologies" is quite confusing.

Reviewer #2 (Remarks to the Author):

I'm satisfied with the responses and revision of the text by the authors, and have no further comments. Therefore, acceptance as its present form is recommended.

Reviewer #3 (Remarks to the Author):

I have no further feedback for the authors, very nice job!

** Visit Nature Portfolio's author and referees' website at www.nature.com/authors for information about policies, services and author benefits**

Open Access This Peer Review File is licensed under a Creative Commons Attribution 4.0 International License, which permits use, sharing, adaptation, distribution and reproduction in any medium or format, as long as you give appropriate credit to the original author(s) and the source, provide a link to the Creative Commons license, and indicate if changes were made.
In cases where reviewers are anonymous, credit should be given to 'Anonymous Referee' and the source.
The images or other third party material in this Peer Review File are included in the article's Creative Commons license, unless indicated otherwise in a credit line to the material. If material is not included in the article's Creative Commons license and your intended use is not permitted by statutory regulation or exceeds the permitted use, you will need to obtain permission directly from the copyright holder.
To view a copy of this license, visit https://creativecommons.org/licenses/by/4.0/

EDITOR COMMENTS

Your manuscript titled "Drought Sensitivity and Diverse Growth Responses of Canadian Forests to Climate Warming" has now been seen by 3 reviewers, and we include their comments at the end of this message. They find your work of interest, but some important points are raised. We are interested in the possibility of publishing your study in Communications Earth & Environment, but would like to consider your responses to these concerns and assess a revised manuscript before we make a final decision on publication. In revision, please fully consider potential factors influencing the tree climate sensitivity, including tree age as suggested by reviewer #2.

We appreciate the thoughtful assessment and comments. We have revised the manuscript accordingly, and we provide a point-by-point response below. Line numbers highlighted in red below refer to corresponding changes in the "track changes" version of the revised manuscript.

REVIEWER COMMENTS:

Reviewer #1 (Remarks to the Author):

This manuscript presents a comprehensive and valuable analysis of tree-growth responses to climate warming across Canadian forests over a 120-year period. The study leverages an extensive tree-ring network to convincingly demonstrate that drought sensitivity, rather than cold limitation, is already the dominant constraint for nearly half of the forested area. The identification of four characteristic temporal response modes (persistent, transitioning, reversible, and buffered) provides a nuanced and important framework for understanding forest dynamics under climate change. The work is data-rich, the methodology is largely sound, and the conclusions have significant implications for predicting forest productivity and carbon sink potential. The manuscript is suitable for publication after addressing some minor points to improve clarity and precision. I therefore recommend Minor Revisions.

Thank you for your favorable review and the comments below.

Title: Drought sensitivity was not quantified separately, but rather falls under the broader category of diverse growth responses to climate. It is advised to adjust the title for more precise expression.

We agree that drought sensitivity was not quantified as a separate metric but rather emerged as one end of the spectrum of growth–climate response types identified in our clustering analysis. We revised the title to read:

"*Shifting Growth–Climate Limitations in Canadian Forests under Observed Climate Change*"

The number of sampling sites described in the abstract and the subsequent results section of the paper is inconsistent. Please double-check this.

Thank you for noting this inconsistency. We double-checked the site count and corrected the abstract to match the Results and Methods. The correct number is 4,179 sites (with 38,395 individual tree chronologies). We added "individual tree" to chronologies where applicable, because we later also calculated and report on "site chronologies".

Line 109: It is recommended to add a cluster analysis of the site chronologies.

Yes, the wording here was misleading. What the reviewer suggest is actually exactly what we did, but this was not clear. Mentioning the number of individual tree chronologies in this place was misleading. The revised section reads:

"*To identify broad modes of climatic control on tree growth, we analyzed correlations between annual ring-width indices and monthly temperature and precipitation. The analysis was carried out for 14, 101 chronologies that represented aggregated site × species × period combinations (a breakdown of sample sizes is provided in Supplementary Table 3)*" (lines 108-111)

The median number of trees included in the site chronologies is 6? Are all the chronologies reliable? Additionally, it is recommended to clarify the reliability of the chronologies across the different time periods analyzed for climate correlation.

Yes, there are many sites in the database that have a small number of trees per site x species, but note that this already represents an aggregation of multiple cores per tree. To improve reliability, we excluded individual tree chronologies with interseries correlation < 0.6. In other words, for sites with low N to be included in site chronology construction, they had to correlate reasonably well. In total, ~4% of individual tree chronologies were removed due to low correlation or data inconsistencies. Site chronologies were then constructed with the remaining cores that correlated well. This also meant that some chronologies with two or few individual tree chronologies were completely removed from further processing.

The next level of quality control was the calculation of mean inter-series correlation (R-bar) and the Expressed Population Signal (EPS) for each site × species × period combinations. We tested various R-bar and EPS thresholds and their effects on the results. The overall results in terms of cluster types and transitions were very stable and did not depend strongly on the exact EPS and R-bar thresholds applied (which is expected with such a large database). In the end we applied an EPS threshold of >0.8 for site × species × period to ensure reliability.

The above is described concisely and comprehensively in the method section, lines 441-456. We are not sure this should be expanded, but we fixed some errors in that section, where we should have referred to "individual tree chronologies" rather than "cores".

The Methods section contains some disorganized and incomplete descriptions of the results. For example, the generation of tree-ring index series, including the detrending methods, should be included in the "Tree-ring data" chapter.

Yes, the reviewer is technically correct in that some of the statistics we obtained in the data processing pipeline could be viewed or reported as results. However, it is not our preference to

do so. From a reader's perspective it is better if the results relate to the stated research objectives rather than to intermediate quality control steps. We could produce appendices of various statistics, but these are routine quality checks in dendrochronology. We decided to keep them briefly described in principle in the methods section.

Reviewer #2 (Remarks to the Author):

This manuscript addresses a critical and timely question regarding the dynamic responses of Canadian forests to climate warming, with a specific focus on the shift between cold and drought limitations. By adopting a comprehensive tree-ring dataset from Canada, the research fills a key gap in understanding how boreal forests that have been traditionally assumed to be cold-limited are increasingly constrained by drought. The large spatial and temporal scope provides robust statistical power to detect regional and temporal patterns of growth-climate relationships. The conclusions are reliable. I have the following major concerns for the authors to improve the manuscript:

Thank you for your thorough review. Please see below how we have addressed your comments and suggestions.

1. Although the authors mention controlling for age effects by comparing consecutive 40-year periods, tree age can independently modulate climate sensitivity (e.g., younger vs. mature trees). The current analysis assumes age effects are consistent across regions and periods, but this assumption is not explicitly tested. I suggest the authors incorporate tree age as a covariate in the cumulative link mixed models to isolate age-related variation from climate-driven shifts in limitation types. Additionally, provide more detailed information on age distributions within and across chronologies to assess potential confounding.

We followed the reviewer's suggestion and re-ran the cumulative link mixed models including chronology age as a covariate. The resulting estimates were virtually identical to those from the original analysis and did not alter the temporal patterns of response types across regions and species (see table below). Age can be an important covariate in dendrology research, but at this level of regional aggregation and continental scale analysis, environmental factors dominate.

Region / Species	AGE as covariate			Original analysis		
	P1	P2	P3	P1	P2	P3
Interior BC						
PINUCON	3.2	2.6	2.5	3.3	2.8	2.7
PSEUMEN	2.8	2.9	2.0	3.0	3.1	2.3
Northwest Territories						
PICEGLA	4.5	2.8	2.5	4.6	3.0	2.7
PICEMAR	4.7	3.1	2.9	4.8	3.3	3.0
PINUBAN	5.0	3.2	2.9	5.1	3.3	3.1
POPUTRE	4.6	4.3	2.2	4.7	4.4	2.4

Northern AB						
PICEGLA	4.1	2.6	2.8	4.1	2.7	3.0
PICEMAR	4.0	3.3	3.2	4.0	3.4	3.3
POPUTRE	3.8	3.3	3.2	3.9	3.3	3.3
Central Boreal						
PICEGLA	2.8	2.6	2.7	2.9	2.7	2.9
PICEMAR	3.7	2.8	3.9	3.8	2.8	4.1
PINUBAN	4.2	2.8	3.4	4.3	2.8	3.6
POPUTRE	4.2	3.5	4.4	4.2	3.6	4.5
Northern ON & QC						
PICEGLA	3.9	3.7	4.2	4.1	4.0	4.5
PICEMAR	5.8	3.7	4.5	6.0	3.9	4.8
PINUBAN	3.8	4.3	4.2	3.9	4.4	4.4
Maritimes						
PICEMAR	5.4	4.9	4.9	5.6	5.1	5.2

Given the near-identical results, we retained the simpler model in the manuscript. We prefer to address confounding effects through careful interpretation (as described in lines 417-427). A covariate cannot help with determining ultimate causes, and the coefficients become less easy to interpret. Our inferences are already strong, relying on paired comparisons of consecutive 40-year periods where age increments are similar across species and regions, which provides a more transparent control for potential age effects.

We added a sentence in the Methods noting that models including chronology age produced comparable results, and placed it after the paragraph where we carefully justify our sampling design in the context of age effects. The additional paragraph reads as follows:

"*To further evaluate potential age-related confounding, we repeated the cumulative link mixed model analyses including chronology age as a covariate. This did not materially change the estimated response-type trajectories across periods, species, or regions. We therefore retained the original model specification. Including age as a covariate does not resolve the confounding between age and time because chronology age increases systematically between consecutive periods. In such cases, regression adjustment cannot separate the effects of two correlated variables. Instead, our inference relies on paired comparisons of consecutive 40-year periods where age increments are similar among regions and species, which provides a transparent control for potential age effects.*" (lines 429-437)

2. The study identifies reversible shifts between drought and cold limitation in the Central Boreal and Northern Ontario & Quebec regions but offers limited mechanistic explanation for these transitions beyond "plastic canopy and hydraulic adjustments." Integrating trait data (either from literature or existing databases) would strengthen the mechanistic link between climate history, plastic adjustments, and growth responses.

We agree that trait-based mechanisms could help explain the reversible shifts between drought and cold limitation. Spatially explicit trait data corresponding to the chronology sites and time periods analyzed are, however, not available for a direct analysis or to directly test this interpretation. Instead, we expanded the Discussion to provide a clearer mechanistic interpretation of these reversals, emphasizing physiological acclimation processes such as adjustments in leaf area, stomatal regulation, carbon allocation, and hydraulic architecture, as well as drought legacy effects documented in recent studies. The revised text and supporting references read as follows:

"*In contrast, forests of the central Boreal and northern Ontario and Quebec showed mid-century shifts toward stronger drought limitation followed by late-century reversals back toward cold-limited responses (Table 2). The reversible shifts likely reflect physiological acclimation rather than direct tracking of climate variables. Trees can adjust leaf area, stomatal regulation, carbon allocation, and aspects of hydraulic architecture in response to repeated drought exposure, thereby modifying the balance between temperature and water limitations on growth*[24]*. Reductions in leaf area or stomatal conductance can lower transpirational demand, while longer-term adjustments in water-transport capacity and allocation may increase hydraulic safety under dry conditions. In addition, such responses may persist beyond the drought event itself as legacy effects, contributing to delayed or reversible changes in growth–climate relationships even under continued warming*[25]" (lines 286-396)

24 Limousin, J. M. *et al.* Drought acclimation of leaves improves tolerance to moderate drought but not resistance to severe water stress. *Plant Cell Environ* **45**, 1967-1984, doi:10.1111/pce.14326 (2022).

25 Kannenberg, S. A., Schwalm, C. R. & Anderegg, W. R. L. Ghosts of the past: how drought legacy effects shape forest functioning and carbon cycling. *Ecol Lett* **23**, 891-901, doi:10.1111/ele.13485 (2020).

3. While the study contrasts generalist vs. specialist species, some widespread species (e.g., black spruce) show mixed responses across regions. The authors attribute this to local conditions, but it would be valuable to explicitly test whether these regional differences correlate with specific environmental gradients (e.g., aridity, soil texture) or genetic variation.

Yes, this is a good suggestion in principle. However, similar to the previous comment, explicitly testing environmental gradients or genetic variation would be difficult to address with the available data. Regional differences in growth–climate responses reflect changes in climate (which we analyze), and their interactions with baseline climate, edaphic conditions or population-level genetic variation. Even if all these variables were available, an empirical analysis would still be limited by confounding among regional differences in climate, soils, and population structure. Disentangling these effects would ultimately require experimental approaches where individual drivers can be manipulated independently. We have clarified this limitation in the Discussion:

"*We should note, however, that regional variation in response types of widespread species may reflect interacting influences of macroclimate, site conditions, and population-level genetic variation, which cannot be disentangled with observational data alone*" (lines 323-326)

4. The study uses CMI to characterize water balance, but the relationship between CMI and actual soil moisture availability (which depends on soil properties and topography) is not explicitly validated. In regions with high baseline precipitation (e.g., the Maritimes), soil moisture buffering may decouple CMI from plant-available water. I suggest the authors include a brief discussion of how soil characteristics or topographic variation might modify the relationship between CMI and drought stress, and note this as a potential source of uncertainty in regions with complex terrain.

This is a valid point and closely related to the previous comment regarding environmental gradients. CMI represents climatic moisture balance and does not explicitly capture local variation in plant-available water arising from soils or topographic water redistribution. While gridded data for such variables exist, their effects would be difficult to disentangle from regional climate differences in an observational analysis. Addressing the underlying mechanisms would ultimately require experimental approaches where individual drivers can be manipulated independently. We added a note in the discussion acknowledging this limitation, extending the discussion point from the previous reply cited above:

"*We should note, however, that regional variation in response types of widespread species may reflect interacting influences of macroclimate, site conditions, and population-level genetic variation, which cannot be disentangled with observational data alone. For example, black spruce commonly occurs on wet or poorly drained sites associated with topographic convergence or soil conditions that promote waterlogging. If such topo-edaphic factors buffer drought responses more strongly in regions such as the Maritimes than elsewhere, they could contribute to the more persistent cold-limited behavior observed there. However, these influences cannot be separated from regional climatic differences, except through experimental research.*" (lines 323-331)

5. The terms "drought limitation" and "drought sensitivity" are used interchangeably throughout the manuscript, but they are not strictly synonymous (sensitivity refers to responsiveness, while limitation implies a constraint on growth). Suggested revision: Standardize terminology to distinguish between sensitivity (correlation with moisture variables) and limitation (growth reduction due to moisture deficit). This clarification would avoid ambiguity, particularly in discussions of species-specific responses.

This is a helpful suggestion. We did not always use the term accurately. We reviewed the manuscript and made corrections as needed. We now use "*sensitivity*" when referring to statistical correlations between tree growth and climate variables, and "*limitation*" when referring to inferred growth constraints associated with those response patterns.

Briefly, we replaced "*sensitivity*" with "*limitation*" in the Abstract, Species response interpretation, Discussion of drought effects. We kept sensitivity in Figure 1, Table 1, Methods, and when describing correlations.

Below are some minor concerns:

1.Figure 2 effectively maps spatial patterns of limitation types, but temporal shifts in response types (e.g., cold-to-drought transitions) are only presented in Table 2, which is dense and difficult to interpret. Please add a supplementary figure (e.g., stacked bar charts or heatmaps) showing temporal changes in response type frequencies for key species-region combinations (e.g., white spruce in the Northwest Territories). This would make temporal trends more accessible to readers.

This is a good idea. We added temporal patterns as Supplementary Figure 1, showing the spatial distributions of response types in each 40-year period. We agree that species-specific frequency plots could also be informative, but given the number of species-region-time combinations, we felt these would be overly fragmented and less interpretable than Table 2.

2.The abstract mentions "four characteristic modes of growth response to climate change," but the results and discussion describe five modes (persistent drought, cold-to-drought transition, reversible shifts, stable cold limitation, and the unique lagged response of high-elevation species). Please clarify this discrepancy to avoid confusion.

Thank you for noting this. The unique lagged response of high-elevation species is not a temporal mode of response to climate change, but rather a distinct response pattern discussed separately. We are almost certain that this confusion was caused by a subtitle in the discussion section that made the distinction of response modes (to climate change) and the discussion of general species- or population specific general response types confusing. This is now fixed with an appropriate discussion subheading:

"*Four characteristic modes of growth response to climate change"* (line 263)

3.In the Methods section, specify the criteria for selecting the seven response clusters (e.g., the scree plot of residual error should be referenced or included as a supplementary figure to justify the cluster number).

The number of clusters was selected based on a scree plot of residual error, following standard practice in multivariate analysis to identify a point of diminishing returns in variance explained. Beyond seven clusters, additional groups did not represent distinct or interpretable response types, but instead subdivided a continuum of growth–climate relationships without adding new insights. We therefore retained seven clusters and clarified this rationale in the Methods. Because this is a routine diagnostic and does not influence the conclusions, we did not include the scree plot in the Supplementary Information. The revised section reads as follows:

"*The number of clusters (n = 7) was chosen based on a scree plot of residual error, identifying a point beyond which additional clusters provided only marginal reductions in residual variance. Within each group, chronologies shared comparable seasonal correlation structures. Additional clusters did not represent distinct or interpretable response types, but rather subdivided a continuum of growth–climate relationships.*" (lines 481-485)

4.The authors note that standardized tree-ring indices were used, which control for growth trends but obscure absolute growth rates. Briefly discuss how this might affect interpretations of productivity changes, as limiting factor shifts do not always translate to absolute growth declines.

Yes, this is an important point. We already note in the Discussion that our analysis is based on standardized tree-ring indices and therefore reflects changes in growth–climate relationships rather than absolute growth. We have slightly expanded this statement to clarify that shifts in limiting factors do not necessarily translate into corresponding changes in absolute productivity, but rather indicate changes in the relative importance of climatic constraints on growth. The expanded section reads:

"That said, these conclusions are based on changes to limiting climatic factors, not absolute growth, since our tree-ring indices were standardized. As a result, shifts in growth limitation do not necessarily translate into corresponding changes in absolute productivity, but rather reflect changes in the relative importance of climatic constraints on growth." (lines 353-357)

Reviewer #3 (Remarks to the Author):

The manuscript by Sang et al., is well written and very interesting. I only have a few minor suggestions for this manuscript.

1. I was a little confused by the seven response clusters of chronologies compared to the ten clusters based on climate trends. I find both of these analyses interesting but was looking for a more in terms of how these two clusters are interacting. The authors talk about this in the text somewhat but I think they could do a better job illustrating how the two clustering schemes interact perhaps in figure 4, which isn't really doing a lot except showing 10 clusters. Can the authors use the 10 climate clusters to help predict the seven response clusters in a model.. perhaps a mixed effect model?

Thank you for pointing this out. We agree that the purpose of the two clustering applications needs to be communicated more clearly. The seven response clusters describe growth–climate relationships derived from tree-ring data, whereas the ten climate clusters represent regional trajectories of climate change over time. In the manuscript, we use the climate clusters like geographic regions (Fig 4) to provide context for interpreting temporal shifts in response types (Table 2).

We clarified this in two places:

"*Chronologies with similar seasonal correlation patterns were grouped into seven response types, ranging from strongly drought-limited to strongly cold-limited. These response types describe growth-climate relationships at the site level and are used throughout the manuscript to characterize patterns of climatic limitation. These groups, hereafter referred to as clusters 1–7, capture the principal axes of forest growth responses to temperature and moisture across Canada*" (lines 112-117)

and:

"*To examine how growth–climate relationships evolved through time, we grouped chronology sites into ten climate-change regions representing coherent trajectories of temperature and moisture change (see Methods). These climate clusters are used as regional groupings (Fig. 4) to summarize shared climate histories and provide context for interpreting temporal shifts in response types, rather than to predict response types directly*." (lines 161-165)

Because the two clustering schemes serve different purposes and because substantial within-region and species-level variability remains, we did not pursue a formal predictive model linking them directly.

2. It would be cool to see what 40-year period the chronologies switched from cold to drought (for those sites that did) on a map… and then see the ones that reversed back to cold and what 40-year period that happened. Perhaps Table 2 was getting at this but not with correlation values… I was also a bit confused by the gray shading. Having the spatial component of table 1 would be nice to see as well.

That's a good suggestion. To better illustrate the timing of shifts in response types, we added a Supplementary Figure 1, showing spatial patterns of response types for each of the three consecutive 40-year periods. This allows visual identification of regions where shifts from cold to drought limitation (and reversals) occurred over time in addition to Table 2.

We also clarified the Table 1 and Table 2 captions with regards to what the shading indicates.
