# Supplementary material for: Shifting growth–climate limitations in Canadian forests under recent climate change
Source: Commun Earth Environ. 2026 Jun 26;7(1):828. doi: 10.1038/s43247-026-03713-7 (PMC13672052; doi:10.1038/s43247-026-03713-7)
Supplement: Supplementary file 2 [file 43247_2026_3713_MOESM2_ESM.pdf]

Supporting Information for: Shifting Growth–Climate Limitations in Canadian Forests under Observed Climate Change

Zihaohan Sang[1*], Andreas Hamann[2], Martin P. Girardin[3], Vinicius Manvailer[4]

Communications Earth & Environment

Content

Supplementary Table 1.

Supplementary Table 2.

Supplementary Table 3.

Supplementary Figure 1.

Supplementary Table 1. Estimates of forested areas (km^2) that are predominantly drought-limited (Clusters 1-4) versus cold limited (Clusters 5-7). The estimates were derived from forest cover of ecodistricts that were classified as on average being drought or cold limited (i.e. corresponding to Fig 2). The total approximately matches the forest cover of 3.47 million km^2 reported by Statistics Canada (2017).

Limiting factor	Forest cover
Drought-limited (Clusters 1-4)	1,614,192
Cold-limited (Clusters 5-7)	342,810
No data (n<10 chronol./ecodist)	1,490,083
Total	3,447,084

Supplementary Table 2. Changes in climate by region as shown in Fig. 4 across three consecutive 40-year periods. Δ12 indicates the change from 1901-1940 to 1941-1980 (values for 1941-1980 subtract the ones for 1901-1940), Δ23 indicates the change from 1941-1980 to 1981-2020. Positive values for MAT indicate warming signal (red); positive MAP or MSP indicate higher precipitation (blue); higher CMI and lower AHM/SHM values indicate greater water availability (blue), while greater dryness indicated in red.

Variable	Period Comparison	Interior BC	Northern AB	Northwest Territories	Central boreal	Northern ON/ QC	Maritime
Temperature (°C)							
MAT	Δ12	+0.28	+0.27	+0.54	+0.27	+0.55	+0.54
	Δ23	+0.64	+0.90	+1.00	+0.84	+0.62	+0.48
Preciptation (%)							
MAP	Δ12	+5.63	+4.14	+8.02	+4.86	+3.03	+3.17
	Δ23	+3.14	+0.00	+3.78	+3.35	+0.42	+1.24
MSP	Δ12	+6.58	+4.39	+4.39	+4.31	+3.38	+2.04
	Δ23	+6.00	+2.79	+9.27	+6.22	+0.29	+3.88
Dryness indices (%)							
CMI	Δ12	+5.44	+1.81	+2.30	+2.68	+2.02	+3.16
	Δ23	+1.42	–0.49	+0.24	+0.77	–1.74	-0.89
AHM	Δ12	–0.77	–0.28	–0.01	–0.49	+0.30	+0.26
	Δ23	+0.18	+1.86	+2.01	+0.98	+0.69	+0.35
SHM	Δ12	–4.51	–1.30	-–2.12	–1.94	-0.80	–0.08
	Δ23	–1.10	–0.71	–5.10	–2.36	+1.24	+0.36

Supplementary Table 3. Number of chronologies used in this analysis after applying various quality control filters and allowing for no more than 10 missing values in a chronology per period. Species are sorted from most to least data in the 1951 to 2000 period.

		Periods for temporal analysis			Period for spatial
Common name	Species	1901-1940	1941-1980	1981-2020	analysis 1951-2000
Black spruce	PICEMAR	1105	1536	1387	1552
Trembling aspen	POPUTRE	144	512	484	536
White spruce	PICEGLA	409	528	364	517
Jack pine	PINUBAN	185	366	302	376
Lodgepole pine	PINUCON	184	195	39	210
Douglas fir	PSEUMEN	164	188	87	135
Balsam fir	ABIEBAL	54	92	85	111
Engelmann spruce	PICEENG	99	103	21	96
Tamarack	LARILAR	50	83	68	93
Paper birch	BETUPAP	35	67	57	75
Subalpine fir	ABIELAS	62	67	20	69
Other species					380
Total number of chronologies		2777	4091	3083	4150

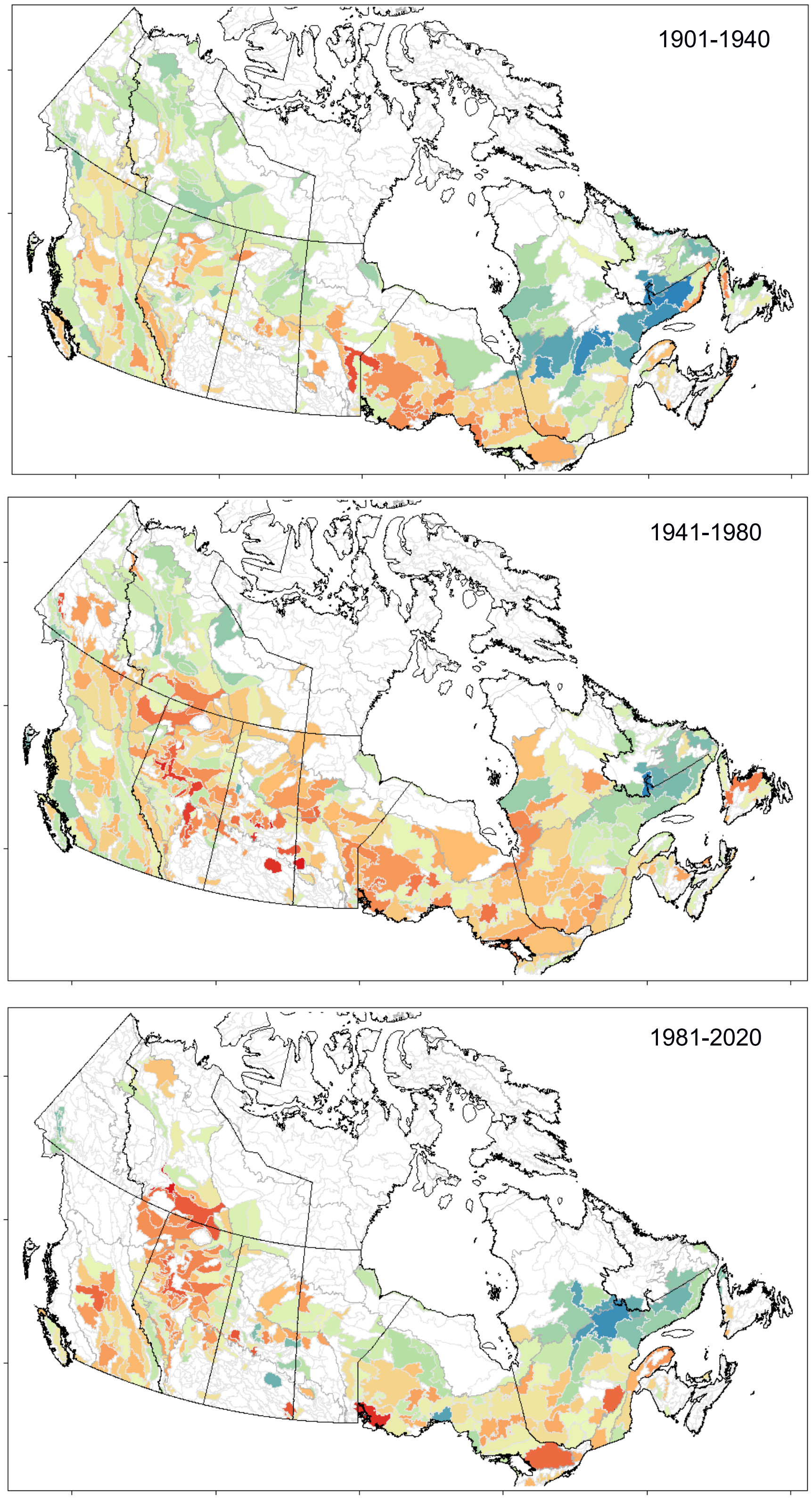

Supplementary Figure 1. Climate sensitivity types broken down by consecutive 40-year periods, color coded from drought limited (red shades) to cold limited (blue shades) as in Figs. 1 and 2 of the article.
